# Microsatellite characterization and marker development for the fungus *Penicillium digitatum*, causal agent of green mold of citrus

**DOI:** 10.1002/mbo3.788

**Published:** 2019-01-30

**Authors:** Erika S. Varady, Sohrab Bodaghi, Georgios Vidalakis, Greg W. Douhan

**Affiliations:** ^1^ Department of Microbiology and Plant Pathology University of California Riverside California; ^2^ Department of Molecular Biology and Biochemistry University of California Irvine Irvine California; ^3^ University of California Cooperative Extension Tulare California

**Keywords:** citrus, fungi, microsatellites, *Penicillium*, population genetics, postharvest

## Abstract

*Penicillium* *digitatum* is one of the most important postharvest pathogens of citrus on a global scale causing significant annual losses due to fruit rot. However, little is known about the diversity of *P. digitatum* populations. The genome of *P. digitatum* has been sequenced, providing an opportunity to determine the microsatellite distribution within *P. digitatum* to develop markers that could be valuable tools for studying the population biology of this pathogen. In the analyses, a total of 3,134 microsatellite loci were detected; 66.73%, 23.23%, 8.23%, 1.24%, 0.16%, and 0.77% were detected as mono‐, di‐, tri‐, tetra‐, penta‐, and hexanucleotide repeats, respectively. As consistent with other ascomycete fungi, the genome size of *P. digitatum* does not seem to correlate with the density of microsatellite loci. However, significantly longer motifs of mono‐, di‐, and tetranucleotide repeats were identified in *P. digitatum* compared to 10 other published ascomycete species with repeats of over 800, 300, and 900 motifs found, respectively. One isolate from southern California and five additional isolates from other countries (“global isolates”) were used to initially screen microsatellite markers developed in this study. Twelve additional isolates, referred to as the “local isolates,” were also collected from citrus at the University of California Riverside agricultural experiment station and were subsequently used to screen the primers that sequenced well and were polymorphic based on the global isolates. Thirty‐six primers were screened, and nine trinucleotide loci and one hexanucleotide locus were chosen as robust markers. These loci yielded two to seven alleles and will be useful to study population genetic structure of *P. digitatum* populations.

## 1 INTRODUCTION

Microsatellites are short tandem repeats of DNA that are ubiquitously distributed throughout the genomes of both eukaryotic and prokaryotic organisms (Tautz & Schlötterer, 1994). Investigations of microsatellite variability have been used to study population genetic differentiation, migration, genetic drift, mutation rates, and other ecological and evolutionary processes that shape the population genetic structures of many types of organisms (Putman & Carbone, 2014). Within the Eukaryotes, studies investigating microsatellite variability within the Fungal Kingdom have lagged compared to both the Plant and Animal Kingdoms in general. Two of the factors that contribute to this are as follows: (a) The mycological community of researchers is small compared to groups studying other organisms such as mammals or plants and (b) distinct microsatellite loci in fungi appear to be much less abundant compared to other organisms studied, and the standard techniques to isolate microsatellites, such as selection using hybridization of specific biotin‐labeled microsatellite motif probes, are difficult to achieve for many fungal species studied thus far (Dutech et al., 2007).

However, full genome sequencing of many different fungal species has enabled mycologists to investigate not only microsatellite distribution within specific fungal genomes but also allowed them to compare this information to other sequenced fungi (Karaoglu, Lee, & Meyer, 2005; Lim, Notley‐McRobb, Lim, & Carter, 2004; Simpson, Wilken, Coetzee, Wingfield, & Wingfield, 2013). Specific sequenced fungal genomes have allowed access to a plethora of potential microsatellites to develop markers to address various questions regarding the population biology of specific fungal species and/or species complexes (Simpson et al., 2013). Moreover, next‐generation sequencing technologies have also become relatively inexpensive, which has allowed researchers the ability to develop microsatellite markers from incomplete genomic data for population‐level studies for nonmodel organisms in general including fungi (Abdelkrim, Robertson, Stanton, & Gemmell, 2009; Cai, Leadbetter, Muehlbauer, Molnar, & Hillman, 2013; Yu, Won, Jun, Lim, & Kwak, 2011).

*Penicillium digitatum* is one of the most important postharvest pathogens of citrus on a global scale, which can be responsible for up to 90% of total crop loss after packing, storage, transportation, and marketing (Eckert, Sievert, & Ratnayake, 1994). *Penicillium digitatum* is a haploid fungus, which belongs to the Phylum Ascomycota, and is only known to reproduce asexually, but is found essentially everywhere citrus is produced. Despite the economic importance of this fungal pathogen, interesting ecology, and global distribution, few studies have been published on the population biology of *P. digitatum*. Most studies on *P. digitatum* diversity have focused on various aspects of fungicide resistance within “agricultural” populations and/or citrus packing plants from various citrus growing regions (e.g., Sánchez‐Torres & Tuset, 2011).

The genome of *P. digitatum* has been sequenced, thus allowing for the mining of this genome for microsatellite markers (Marcet‐Houben et al., 2012). Therefore, the objectives of this study were to (a) determine the distribution of microsatellite loci in the published genome of *P. digitatum* and (b) to design primers from specific loci to develop markers that would have utility for future population genetic studies of this important pathogen. To accomplish this, a global representation of isolates of *P. digitatum* was used to screen various loci to determine whether variation could be found by sequencing each locus. Variable loci were then further screened from a local California collection of isolates to gain insight into the utility of the markers for both fine‐scale and global perspectives.

## 2 MATERIALS AND METHODS

### 2.1 Microsatellite characterization and analyses

The genome of *P. digitatum *was downloaded from the Joint Genomics Institute (JGI); http://genome.jgi.doe.gov/Pendi1/Pendi1.home.html. The genome of *P. digitatum *used in this study was published by Marcet‐Houben et al. (2012) from an isolate (PHI126) recovered from orange in Valencia, Spain. The final assembly of the genome resulted in a genome size of approximately 26 Mb with an average GC content of 48.9%. The program Msatcommander 1.0 (Faircloth, 2008) was used to characterize the entire mono‐, di‐, tri‐, tetra‐, penta‐, and hexanucleotide microsatellites within the genome of *P. digitatum* isolate PHI126. The parameters set were to restrict mononucleotide repeats to 12 bp and above, while the rest were restricted to five repeats and above. The output files from Msatcommander 1.0 were sorted in Microsoft Excel 2016, and the results were compared to other published genomes of ascomycete fungi (Karaoglu et al., 2005; Lim et al., 2004; Simpson et al., 2013). For this comparison, the original data from the published manuscripts were used so the comparisons are relative because slightly different parameters and different software were used to calculate microsatellite density between the different studies. The length distribution of mononucleotide repeats was also compared using SigmaPlot software (Systat Software, San Jose, CA).

### 2.2 Microsatellite search and primer design

Primer pairs for di‐, tri‐, tetra‐, penta‐, and hexanucleotide loci (*n* = 43) were designed using the online version of Primer3 (Koressaar & Remm, 2007; Untergasser et al., 2012) with the default settings. The microsatellite motifs were identified by searching the genome for various repeats and choosing repeats that were at least greater than 9. Only perfect microsatellite loci within the target genome (isolate PHI126) were used to design primers. The flanking regions were also scanned for potential repetitive elements directly outside of the perfect repeats. When a locus was found acceptable, approximately 100–200 bp on either side of the repeat was included to find robust loci with annealing temperatures of at least 60°C. All primers were purchased from Integrated DNA Technologies, Coralville, Iowa. Our approach was to bias toward trinucleotide repeats and above given the difficulty to differentiate dinucleotide repeats using fluorescently labeled primers and capillary sequencing methodologies.

### 2.3 Fungal isolates

To screen the initial primers, five *P. digitatum* isolates, which were originally isolated from South Africa, Uruguay, Argentina, Chile, and Cyprus, were acquired in cetyltrimethylammonium bromide (CTAB) extraction buffer from Dr. Mareli Kellerman, at Stellenbosch University. One North American isolate, collected at the Agricultural Operations (AgOps) facility at the University of California Riverside, was also included in the initial primer screen; the six isolates are referred to as the “global isolates” within this manuscript. Twelve additional isolates, referred to as the “local isolates,” were also collected at AgOps that were subsequently used to screen the primers that sequenced well and were polymorphic based on the global isolates. The local isolates were collected from fallen diseased citrus fruit collected approximately 3–1,400 meters apart from one another. To isolate the fungi, spores were swabbed directly from colonized fruit in the field and placed in sterile H_2_O and dilution plated onto potato dextrose agar (PDA; Becton, Dickinson and Company Franklin Lake, NJ). The cultures were incubated at 25°C for 1–2 weeks. The leading edge of a single colony was then transferred to a new agar plate, incubated for an additional 1–2 weeks, and then, the spores and mycelium were scraped off the agar into CTAB extraction buffer (Gardes & Bruns, 1993).

### 2.4 DNA extraction and microsatellite amplification

DNA extraction was performed on all *P. digitatum* isolates using a slightly modified chloroform/CTAB DNA extraction method of Gardes and Bruns (1993). The genomic DNA was electrophoresed in 0.8% gels and visualized as described below. The DNA was diluted 1:25 in sterile water and 2 μl used as template for all subsequent polymerase chain reaction (PCR) amplifications. PCR amplification of each 20 μl reaction was carried out with a final concentration of 1X PCR buffer, 2.5 mM MgCl_2_, 2.5 mM of each dNTP, 0.5 U of Taq polymerase (Invitrogen, Carlsbad, CA), and 3.75 μM of each primer. PCR amplification conditions were 94°C (3 min), followed by 30 cycles of 94°C (30 s), 60–62°C (30 s), and 72°C (1 min). All PCR amplifications were performed using a Bio‐Rad MyCycler (Hercules, CA). One μl of a 1:100 dilution of SYBR Green (Invitrogen, Carlsbad, CA) was added to 3 μl of PCR product and electrophoresed for 15–20 min at 110 V in 1.5% agarose gels using TBE buffer (1.35 M Tris/HCl pH 8.0, 0.45 M boric acid, 25 mM EDTA). The gels were visualized using a Bio‐Rad Chemidoc system (Hercules, CA).

PCR products were purified using a solution of 1% exonuclease I (10 U/μl), 10% phosphatase (1 U/μl; Affymetrix, Santa Clara, CA), and 89% sterile H_2_O. To purify the PCR products, 1.5 μl of this cocktail was added to 6 μl of PCR product and incubated in a Bio‐Rad MyCycler with the conditions of 1 cycle of 37°C (15 min) and 80°C (15 min). The purified PCR products were sequenced using Sanger sequencing at the Institute of Integrative Genome Biology Genomics Core facility at the University of California Riverside. During the first screening, only a single primer was used and when loci were identified that had clean reads for all tester isolates under our conditions, the other primers were then used to acquire the opposite reads. Contigs were edited using Sequencher software (Gene Codes Corporation, Ann Arbor, MI, USA). The sequences were aligned using ClustalX (Thompson, Gibson, Plewniak, Jeanmougin, & Higgins, 1997) and edited (allele counting) using MacClade (Maddison & Maddison, 2005).

### 2.5 Data analysis

The program POPGENE was used to calculate allele frequencies and various population genetic summary statistics to compare the global and local isolates (Yeh, Yang, Boyle, Ye, & Mao, 1997). Randomization procedures in FSTAT were also used to test for population differentiation between the local and global samples by comparing the allele frequencies using Weir and Cockerham's population differentiation statistic θ (Goudet, 2002). The estimated θ values were tested under the null hypothesis of no differentiation among the global and local isolates by comparing the observed values of θ to values estimated for data sets in which alleles were resampled without replacement 10,000 times (Goudet, 2002). To determine multilocus genotypes (MLG), the data were sorted in Microsoft Excel 2016.

## 3 RESULTS

### 3.1 Microsatellite characterization

**Figure 1:**
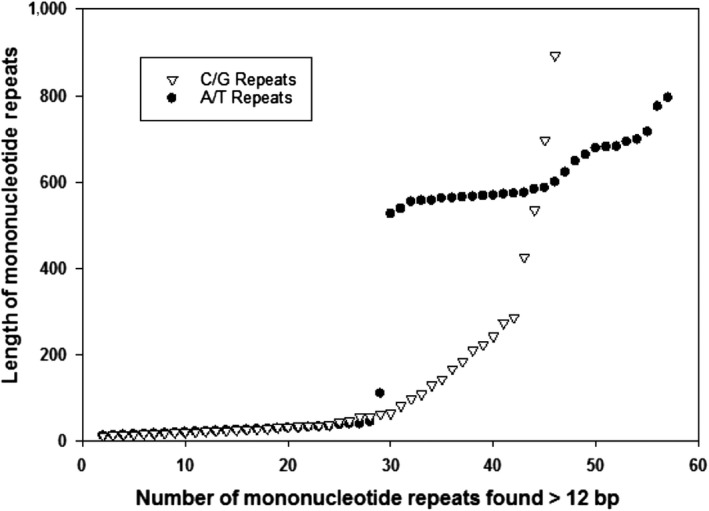
Length distribution of mononucleotide repeats within the genome of *Penicillium digitatum* isolate PHI126

A total of 3,134 microsatellite loci were found in the genome of *P. digitatum *isolate PHI126 based on the parameters used in Msatcommander 1.0 (Table 1). A total of 2,080, 728, 258, 39, 5, 24 mono‐, di‐, tri‐, tetra‐, penta‐, and hexanucleotide repeats were identified, respectively (Table 1). Mono‐, di‐, and trinucleotides were more common than tetra‐, penta‐, and hexanucleotides; these former motif types also had a much greater range of repeat lengths and were found more frequently (Table 1). As with the other ascomycete fungi compared to *P. digitatum*, mononucleotides were the most abundant repeats with a general trend of becoming less abundant as the repeat length gets larger (Table 2). The longest microsatellites found were monomorphic repeats with lengths up to 795 and 893 bp for A/T and C/G repeats, respectively, and there were many long (>200) mononucleotide repeats within the *P. digitatum* genome (Figure 1). When comparing the lengths of the longest repeats of microsatellites published for ascomycete fungi, *P. digitatum* had significantly longer mono‐, di‐, and trinucleotide repeats (Table 3).

**Table 1:** Summary of microsatellite distribution within *Penicillium digitatum *isolate PHI126

Repeat type	Motif	Range of repeats found	No. of times found within range	Total no. of motifs found	Total no. of repeat types
Mono	A	12–795	383–1	1,355	
Mono	C	12–893	111–1	725	2,080
Di	AC	5–290	62–1	120	
Di	AG	5–307	258–1	467	
Di	AT	5–13	88–1	122	
Di	CG	5–8	17–1	19	728
Tri	AAC	5–128	6–1	16	
Tri	AAG	5–17	41–1	101	
Tri	AAT	5 & 8	1	2	
Tri	ACC	5–11	8–1	15	
Tri	ACG	5 & 6	1	2	
Tri	ACT	5–8	2–1	5	
Tri	AGC	5–8	10–1	20	
Tri	AGG	5–13	8–1	22	
Tri	ATC	5–946	23–1	60	
Tri	CCG	5–8	9–1	15	258
Tetra	AAAC	5	2	2	
Tetra	AAAG	5 & 6	3–6	9	
Tetra	AACC	5	1	1	
Tetra	AACT	5–8	1–2	4	
Tetra	AATC	5 & 6	2–5	7	
Tetra	ACAG	5	1	1	
Tetra	ACAT	7	1	1	
Tetra	ACCT	5–10	1–7	11	
Tetra	AGAT	5	2	2	
Tetra	AGGG	5	1	1	39
Penta	AGATG	15	1	1	
Penta	AGGGG	5	1	1	
Penta	AAAAG	5 & 8	1	2	
Penta	AAATG	7	1	1	5
Hexa	AAAACG	6	1	1	
Hexa	AACCAG	7	1	1	
Hexa	AACCGC	5	1	1	
Hexa	AACTCC	5	1	1	
Hexa	AAGAGG	7	1	1	
Hexa	AATAGG	5	1	1	
Hexa	AATATG	6	1	1	
Hexa	AATCAC	5	1	1	
Hexa	ACAGAG	7	1	1	
Hexa	ACAGGC	5	1	1	
Hexa	ACCAGC	5	5–7	3	
Hexa	ACTCAG	5	5 & 6	2	
Hexa	ACTGGC	5	1	1	
Hexa	AGATGC	5	1	1	
Hexa	AGATGG	5	2	2	
Hexa	AGCAGG	5	5 & 6	2	
Hexa	AGCTCC	5	1	1	
Hexa	AGGATG	7	1	1	24
				Grand total	3,134

**Table 2:** Comparison of the microsatellite differences between various ascomycete fungi compared to *Penicillium digitatum *isolate PHI126

Organism	Mono	Di	Tri	Tetra	Penta	Hexa
*Ashbya gossypii* b	97.80	1.40	0.75	0.01	0.01	0.03
*Aspergillus fumigatus* b	97.90	1.35	0.66	0.05	0.02	0.04
*Aspergillus nidulans* a	51.80	31.20	13.50	1.49	1.16	0.79
*Candida albicans* b	95.40	1.62	2.48	0.28	0.08	0.09
*Fusarium graminearum* a	38.30	35.70	19.20	2.94	2.52	1.38
*Magnaporthe grisea* a	69.10	14.90	13.50	1.88	0.28	0.39
*Neurospora crassa* a	41.50	22.40	28.50	5.29	1.34	0.94
*Saccharomyces cerevisiae* a	65.30	22.60	11.00	0.39	0.25	0.53
*Schizosaccharomyces pombe* a	72.20	20.50	6.06	0.65	0.56	0.09
*Ceratocystis fimbriata* c	44.50	34.70	14.60	3.09	1.63	1.50
*Penicillium digitatum*	66.37	23.23	8.23	1.24	0.16	0.77

a Results from Karaoglu et al. (2005).

b Results from Lim et al. (2004).

c Results from Simpson et al. (2013).

**Table 3:** Comparison of the longest microsatellite repeats between various ascomycete fungi compared to *Penicillium digitatum *isolate PHI126

Organism^a^	Mono	Di	Tri	Tetra	Penta	Hexa
*Ashbya gossypii* b	(A)_57_	ND	(AAC)_21_	ND	(AAAAT)_23_	ND
*Aspergillus fumigatus* b	(A)_95_	(AG)_26_	(AAG)_28_	(AAAG)_28_	ND	(AACCCT)_28_
*Aspergillus nidulans* a	(T)_94_	(GT)_36_	(TGA)_31_	(AAAT)_13_	(AAACG)_14_	(TTAGGG)_22_
*Candida albicans* b	(A)_35_	ND	(AAT)_30_	(AAAT)_29_	ND	(AGAGCC)_20_
*Fusarium graminearum* a	(T)_41_	(CT)_28_	(GAA)_46_	(CTTT)_13_	(GTATG)_18_	(TGAAGA)_22_
*Magnaporthe grisea* a	(T)_59_	(GA)_92_	(TGG)_37_	(TACC)_48_	(GGCAA)_29_	(GCCTGA)_58_
*Neurospora crassa* a	(T)_89_	(TC)_78_	(TTA)_93_	(AGGA)_51_	(AAGGA)_32_	(AGGGTT)_28_
*Saccharomyces cerevisiae* a	(T)_42_	(GA)_32_	(TAT)_36_	(AAAT)_13_	(GATGA)_7_	(TGTTTT)_8_
*Schizosaccharomyces pombe* a	(T)_39_	(TG)_19_	(CAA)_28_	(TAAA)_7_	(TATTT)_9_	(ATTATC)_6_
*Ceratocystis fimbriata* c	(G)_62_	(GA)_41_	(AAG)_19_	(TCAC)_15_	(GACAG)_18_	(GAAAAT)_14_
*Penicillium digitatum*	(C)_893_	(AG)_307_	(ATC)_946_	(ACCT)_10_	(AGATG)_15_	(AACCAG)_7_

Note

ND: no data.

a Results fromKaraoglu et al. (2005)

b Results fromLim et al. (2004)

c Results fromSimpson et al. (2013)

### 3.2 Microsatellite variability

Forty‐one out of 43 primers tested were successfully amplified from all of the initial global tester isolates based on the PCR parameters used for this study. Two loci, 3–6 and 3–23, amplified but not from all tester isolates despite multiple attempts. From the initial sequencing results, eight loci were not variable and 25 loci produced sequencing results that could not be easily scored from all tester isolates despite multiple attempts to produce “clean” reads (Appendix 1). A total of 10 loci were further characterized among the loci that yielded quality sequencing reads from both the global and local isolates (Table 4).

**Table 4:** Primer information and genetic diversity statistics for the global and local isolates of *Penicillium digitatum *used in this study

Locus	Accession no.	Motif	Primers (Tm = 62°C)	Global samples (*N* = 6)				Local samples (*N* = 12)			
				Specific repeats	*N_A_*	*h*	*I*	Specific repeats	*N_A_*	*h*	*I*
3‐2	MH375782	GAT_12_	F‐CCTGAAGATCAGGGTGAGGA R‐CTCGGGATCCTCGTTAATCA	GAT_7, 12, 14_	3	0.500	0.868	GAT_7, 12, 13_	3	0.292	0.566
3‐3	MH375783	TTC_18_	F‐CAATATTAAGCCCCGACGAA R‐TCCATCGGCACACTATACCA	(TTC)_46_(TCC)_2_; (TTC)_45_(TCC)_3_; (TTC)_23_(TCC)_3_	3	0.500	0.868	(TTC)_23_(TCC)_3_;(TTC)_45_(TCC)_3_; (TTC)_44_(TCC)_3_; (TTC)_46_(TCC)_3_; (TTC)_47_(TCC)_3_;(TTC)_48_(TCC)_2_; (TTC)_48_(TCC)_3_	7	0.792	1.748
3‐5	MH375784	CTT_13_	F‐GCTGCACTTTGCAACAAAAA R‐CAAAAAGCAAGCCGAAAAAC	CTT_9, 17, 18_	3	0.500	0.868	(CTT)_9, 17_	2	0.153	0.287
3‐8	MH375785	TTC_13_	F‐TCGGCTTCTGCTATTGGTCT R‐GATTATACAGCCGCGACACA	TTC_22, 26, 27_	3	0.500	0.868	TTC_20, 21, 22, 26_	4	0.514	0.983
3‐12	MH375778	AAC_128_	F‐GCATTATAGACGGGGCAGAG R‐AGATGATTCGGTTCGGGAAT	AAC_22, 23, 27_	3	0.500	0.868	AAC_22, 23, 24, 34_	4	0.625	1.127
3‐14	MH375779	ACC_11_	F‐CGTCAAAAGACCACGACTGA R‐GTATCCCTGGTGGCATGG	ACC_9, 11_	2	0.444	0.637	ACC_9, 11_	2	0.153	0.287
3‐15	MH375777	GTT_13_	F‐TCTGCCTCGTACTGATTTTGC R‐CAGCAACAGCAACAACTGCT	GTT_17,18_	2	0.278	0.451	GTT_9, 15, 16, 17_	4	0.514	0.983
3‐16	MH375780	GTT_13_	F‐TTTTGGAGAACATTTGCAACC R‐CAACAAGCCACCTATCCTCCT	GTT_21, 28, 34_	3	0.500	0.868	GTT_19, 20, 27, 28_	4	0.417	0.837
3‐20	MH375781	TGG_10_	F‐ATCATGCCTGAGGAGGACAA R‐AGCTCGCCGTCAGTGATATT	TGG_9, 13, 14_	3	0.500	0.868	TGG_13, 14_	2	0.278	0.451
6‐1	MH375786	ATATGA_10_	F‐GGACTGCAAGGAAACAGAGC R‐TCCCATCCAGCTATGACACA	ATATGA_9, 12, 15_	3	0.500	0.868	ATATGA_5, 7, 9, 12, 14_	5	0.764	1.517

Note

*H*: ei's gene diversity (Nei, 1972); *I*: Shannon information index (Lewontin, 1972); *N_A_*: observed number of alleles.

Nine trinucleotide loci and one hexanucleotide microsatellite locus were characterized that yielded two to three and two to seven alleles within the global and local isolates, respectively (Table 4). Nine out of the 10 loci were perfect repeats, whereas locus 3–3 was found to be a compound repeat in the isolates tested while this locus was a perfect repeat within the sampled genome. Additionally, this repeat in the reference Spain isolate was also only 18 repeats but was significantly longer within the isolates that were sequenced in this study. The opposite, with respect to length differences, were found in locus 3–12 where the repeat length in the Spain isolate was 128 repeats but were considerably shorter in the isolates sequenced. Comparisons of the flanking sequence regions of all sequenced microsatellite loci with that of the Spanish isolate confirmed that the characterized sequenced microsatellite loci were all homologous.

Comparing the global to local isolates, there were also considerable differences with respect to allele frequencies (Table 5) and other summary statistics (Table 4). Obvious differences in allele frequencies between the global and local “populations” can readily be seen in Table 5 as well as many private alleles which resulted in significant population differentiation based on the Fst analysis (*p* < 0.001). Larger number of alleles and higher estimates of diversity were also found within the local isolates compared to the global isolates (Table 4).

**Table 5:** Allele frequencies between the local and global “populations” of *Penicillium digitatum* isolates used in this study

Locus: 3‐2			Locus: 3‐3			Locus: 3‐5			Locus: 3‐8			Locus: 3‐9		
Allele	Local	Global	Allele	Local	Global	Allele	Local	Global	Allele	Local	Global	Allele	Local	Global
7	0.083	0.667	1	0.083	0.667	9	0.083	0.667	20	0.667	0.000	22	0.083	0.667
12	0.833	0.167	2	0.25	0.000	17	0.917	0.167	21	0.167	0.000	23	0.500	0.167
13	0.083	0.000	3	0.333	0.000	18	0.000	0.167	22	0.083	0.167	24	0.333	0.000
14	0.000	0.167	4	0.083	0.000				26	0.083	0.667	27	0.000	0.167
			5	0.083	0.000				27	0.000	0.167	34	0.083	0.000
			6	0.083	0.167									
			7	0.083	0.000									
			8	0.000	0.167									

Locus: 3‐11			Locus: 3‐17			Locus: 3‐12			Locus: 3‐14			Locus: 6‐1		
Allele	Local	Global	Allele	Local	Global	Allele	Local	Global	Allele	Local	Global	Allele	Local	Global
9	0.083	0.667	9	0.000	0.167	9	0.083	0.000	19	0.083	0.000	5	0.167	0.000
11	0.917	0.333	13	0.167	0.667	15	0.083	0.000	20	0.75	0.000	7	0.167	0.000
			14	0.833	0.167	16	0.667	0.000	21	0.000	0.167	9	0.083	0.667
						17	0.167	0.000	27	0.083	0.000	12	0.25	0.167
						18	0.000	0.167	28	0.083	0.667	14	0.333	0.000
						19	0.000	0.833	34	0.000	0.167	15	0.000	0.167

Out of the 18 isolates genotyped based on direct sequencing, only four clonal genotypes were found which were from the global sample of isolates, Chile, Uruguay, Argentina, and Cyprus. The California and South African isolates used in the initial screening accounted for the variable loci observed prior to screening the local isolates. All of the other isolates represented unique MLG, and no single locus was monomorphic, even in this limited sampling of isolates used to develop these markers.

## 4 DISCUSSION

This is the first study to investigate microsatellite distribution within the genome of *P. digitatum* and to develop a set of 10 microsatellite markers for this important global pathogen of citrus. Overall, variation was easily detected within *P. digitatum*; that is, only eight out of the 43 loci were monomorphic. This finding was unexpected given that these fungi are thought to be exclusively asexual species of fungi, yet all MLG found within the local population were unique, even isolates collected ~3 m from one another. These markers can now be utilized in future studies to investigate diversity, population structure, the potential for recombination/sexual reproduction, and other ecological and evolutionary processes that shape *P. digitatum *populations.

Within the genus *Penicillium*, the only two studies to our knowledge to develop microsatellite markers have been for *P. marneffei *(Lasker & Ran, 2004) and *P. roqueforti* (Ropars et al., 2014). *Penicillium marneffei* is a human pathogen, and Lasker and Ran (2004) developed these markers to assist in epidemiological studies for this pathogen. However, *P. marneffei* is actually taxonomically distinct from the genus *Penicillium *and is more closely related to the genus *Talaromyces* (LoBuglio & Taylor, 1995). *Penicillium roqueforti* is the famous fungal species used to produce the marbled effect and taste of blue cheese. In the latter study, Ropars et al. (2014) found significant microsatellite variation from over 100 isolates of *P. roqueforti* and actually induced viable sexual structures of this pathogen in vitro. This was unexpected because these species, like many other *Penicillium *species, are thought to be strictly asexual.

In this study, we also found significant variation, which is consistent with the potential for this species to sexually reproduce which has not been demonstrated. Most isolates were unique MLG, which is especially interesting from the local population that was collected from one general location. Similar results were found by Lee (2002) who only found two clonal isolates out of a 100 sampled primarily between the USA and China using AFLP. In contrast, Julca, Droby, Sela, Marcet‐Houben, and Gabaldón (2015) found very little single nucleotide polymorphisms between global isolates at the genome level of *P. digitatum* and suggested that this species had a recent population expansion as a single lineage. However, this study was only based on four strains of *P. digitatum* with two of them coming from Spain and the others from China and Israel which could have significantly influenced the results of their study. In this study, four of the “global” isolates were clones based on our makers (three from South America and one from Cyprus), consistent with Julca *et al.* (2015). In contrast though, as previously mentioned, most of the isolates were unique MLG. However, global distribution of citrus fruits over decades could easily move clonal genotypes of this postharvest pathogen, but not enough studies have been conducted with adequate sampling sizes to draw any solid conclusions regarding phylogeographic inferences of this species.

In many ascomycete fungi, two mating types (MAT1‐1 and MAT1‐2) are needed in order for sexual reproduction to occur. Some species only have a single mating type within distinct individuals and must outcross, whereas some species can have both mating type genes within a single individual and can self‐reproduce. Within *P. digitatum*, Marcet‐Houben et al. (2012) identified a conserved mating type gene (MAT1‐1) within the genome, and not a MAT1‐2 homologue which suggests that this species may need to outcross if sexual reproduction is possible. Therefore, based on our results of significant variation even at a fine sampling scale, there is a potential that *P. digitatum* may reproduce sexually but the sexual phase has yet to be discovered. This is an intriguing hypothesis but will take further studies to investigate this possibility.

Microsatellite repeats exist in other fungal species and generally share the same pattern in which shorter repeats are more common and variable than larger repeats (Karaoglu et al., 2005; Lim et al., 2004; Simpson et al., 2013), which was also found in this study. In comparison with 10 other ascomycete fungi, *P. digitatum *had significantly longer microsatellite motifs for mono‐, di‐, and trinucleotide repeats (Lim et al., 2004). Moreover, as consistent with other ascomycete fungi, the genome size of *P. digitatum *does not seem to correlate with the density of microsatellite loci. What was unusual in this study compared to other published studies on microsatellites were the extremely large repeats of three loci in the published *P. digitatum* genome. It appears that microsatellites with large numbers of repeats, or long microsatellites, are rare in fungal genomes compared to the human genome (Dutech et al., 2007). However, the biological importance regarding these large repeats is essentially unknown at this time.

For this study, the global isolates were artificially pooled to represent a population so that population differentiation could be compared to the local population. Based on allele frequencies alone (Table 5), it was clear that the two “populations” were differentiated which was confirmed statistically (*p* < 0.001). Many private alleles were also found between both “populations” and when shared alleles were found, the frequency for most of them was very different. Further studies sampling “local” populations throughout citrus growing areas will help to elucidate the population structure of this pathogen and may provide insight into the importance of the mechanism(s) of reproduction and spore movement.

Supplemental data for primers that amplified but could not be sequenced reliably are also provided (Appendix 1). Most of these loci produced clean PCR products; however, it was not possible to get a clean sequencing reads from all six tester isolates, but variation within loci was observed. The microsatellite loci that were unreadable were sequenced at least two times and each time similar unclear reads were obtained. The difficulty of performing Sanger sequencing on microsatellites could be due to the instability of long stretches of nucleotide repeats which has been well documented (Wierdl, Dominska, & Petes, 1997). We took a conservative approach and sequenced until we found 10 loci that could be scored unambiguously. However, these additional loci may also be useful in the future using size selection via fluorescently labeled primer methods as we plan to do for future work to study this important pathogen of citrus.

## CONFLICT OF INTEREST

Authors declare no conflict of interest.

## AUTHORS CONTRIBUTION

E.S.V. and G.W. D. conceived and designed this study, collected and analyzed data, and drafted the article. S. B. and G. V. helped in collecting and analyzing data and critically revised the article.

## ETHICS STATEMENT

Not required.

## Data Availability

Data and respective GenBank accession numbers are provided in Results section.

## APPENDIX 1 Primers for microsatellite loci that amplified but clean sequencing reads were not 100% successful for all six tester isolates.

**Table:**

Locus	Motif	Primers (Tm =62°C)	Locus	Motif	Primers (Tm =62°C)
3‐9	CAT_13_	F‐TGTAGCATCCACTGGTCTCG	2‐3	GA_18_	F‐GGCGCAAATGAATAACCTTG
		R‐AAGCGGAAATTCGAGACAGA			R‐AAGCGGAAATTCGAGACAGA
0	AGA_13_	F‐TGGCTTCAATTCCACTCTTG	2‐4	GT_17_	F‐AGGGAGTAAAGCCCCTGTGT
		R‐GTCTGGGGCAAATGGTAAAA			R‐CAATTCGTCTGTGACGTTG
3‐11	CTC_16_	F‐AACATTGGTCAAACGTGGGTA	2‐5	GA_15_	F‐TGCCAACTCTATTCCCGACT
		R‐TGGATGAGGATGCTCGAGTT			R‐TATGGCTGCACGTGTTTGTT
3‐13	GAA_13_	F‐GAAGGAAAGGGGAAGTGCAT	2‐6	CT_307_	F‐TGCACCGAAACGTGTTACAA
		R‐GGATGCTGAACATCGTTGAC			R‐TCAATGATCCGCCTCTTTTT
3‐17	CAG_9_	F‐CTGGGCTCTGTTGATGTGG	2‐7	TC_186_	F‐AGGACGTACCCAATTCCACA
		R‐TCAGTCTCTTTGGCGAGTTCT			R‐CAAATGGAGGTGCTACTTG
3‐18	ATC_10_	F‐GGGAATGCACCACCATTTAT	3‐1	GGA_11_	F‐AAGAGCGTATCGGACTGGTG
		R‐TTTCTCTTCATCCACCCATT			R‐CCCAACTCCCTACCTCACCT
3‐19	ACC_11_	F‐CATCGTCAAAAGACCACGAC	3‐4	GAA_14_	F‐GTCTAGGGAACCACGGAGTG
		R‐GTATCCCTGGTGGCATGG			R‐TGGAGGTTCGTGTCGTGTA
3‐21	TCT_11_	F‐TCTAGCAGCGATGTGTCTGG	3‐6	ACA_128_	F‐CAGTGTGAGGGGGTGAGACT
		R‐GGACGTCGACGAATAACACC			R‐TATGAGCGGGACTGTTGTG
3‐22	AGG_13_	F‐CCATGCTGTACGATGGCTAA	3‐7	TGA_946_	F‐CCTGCCTTACCTCAGATCCA
		R‐GGGCTACAAGATTGCTGGAA			R‐GACCTCGGACTTGATCTCCA
3‐23	TGG_10_	F‐ATGATCCTCTGGCCTATGCT	5‐1	TGAGA_15_	F‐ACCCCTGTTAGCTCTGCAAT
		R‐CGCCTAGCATACCAGTCCA			R‐GATCAGCTCAGCACCACAT
4‐1	GTAG_11_	F‐CAGGAACCTTGGTTTTTGTCA	5‐2	GAAAA_8_	F‐AGCGTGATTCCTCCGGTATT
		R‐TAGCACTGCACGTCCCAATA			R‐ACTGGCCGCAAATTGGATG
2‐1	AG_22_	F‐ACTATTTCGGGACAGCGTCT	5‐3	TTCAT_7_	F‐CTGAAGCAAAGCTTGGGAAC
		R‐ATTCATTGGTGGCTTTGGT			R‐ACATGGAACCTTCCCTCTG
2‐2	AG_20_	F‐CCCGAATCTATCGAGTTTGC			
		R‐CACATCCCGCTTTTCCTTT			
